# Use of thulium-sensitized rare earth-doped low phonon energy crystalline hosts for IR sources

**DOI:** 10.1186/1556-276X-8-455

**Published:** 2013-11-01

**Authors:** Joseph Ganem, Steven R Bowman

**Affiliations:** 1 Department of Physics, Loyola University Maryland, 4501 N. Charles Street, Baltimore, MD 21210, USA; 2 Optical Sciences Division, Naval Research Laboratory, 4555 Overlook Avenue SW, Washington, DC 20375, USA

**Keywords:** Rare earth-doped crystals, Thulium cross-relaxation, Solid-state optical cooling, Mid-infrared sources, Optically pumped mid-infrared phosphors, Potassium lead chloride, 42.72.Ai, 78.60.Lc, 63.20.kd

## Abstract

Crystalline hosts with low phonon energies enable novel energy transfer processes when doped with rare earth ions. Two applications of energy transfer for rare earth ions in thulium-sensitized low phonon energy crystals that result in infrared luminescence are discussed. One application is an endothermic, phonon-assisted cross-relaxation process in thulium-doped yttrium chloride that converts lattice phonons to infrared emission, which raises the possibility of a fundamentally new method for achieving solid-state optical cooling. The other application is an optically pumped mid-IR phosphor using thulium-praseodymium-doped potassium lead chloride that converts 805-nm diode light to broadband emission from 4,000 to 5,500 nm. These two applications in chloride crystals are discussed in terms of critical radii calculated from Forster-Dexter energy transfer theory. It is found that the critical radii for electric dipole-dipole interactions in low phonon energy chloride crystals are comparable to those in conventional oxide and fluoride crystals. It is the reduction in multi-phonon relaxation rates in chloride crystals that enable these additional energy transfer processes and infrared luminescence.

## Background

Rare earth-doped crystals are widely used in many applications that require sources of visible and near-infrared radiation. However, when doped into conventional commercially available crystals such as YAG or YLF, rare earth ions do not radiate efficiently at wavelengths much longer than 3 μm. The mid-infrared range (3 to 10 μm) is not directly accessible using host crystals that have tightly bound oxygen or fluorine ions. The reasons are the relatively high energies for lattice phonons in these crystals and the fact that the rates for non-radiative multi-phonon relaxation increase exponentially as the energies of the electronic transitions are reduced and fewer phonons are required to bridge the gap. The demand for mid-infrared sources and applications in gas detection, remote sensing, IR spectroscopy, and infrared countermeasures has motivated research on alternative methods for generating mid-infrared. Quantum cascade lasers [1], thermal tungsten filaments, small bandgap III-V or II-VI optically pumped semi-conductors [2,3], rare earth-doped chalcogenide glasses [4], oxide glasses [5], and rare earth-doped fluoride crystals [6] have all been used as sources of mid-infrared. This paper discusses an approach to generating mid-infrared that uses rare earth-doped crystals with reduced phonon energies. It focuses specifically on crystals sensitized for diode pumping with the trivalent rare earth ion thulium (Tm^3+^). This ion is interesting because it has a strong absorption near 800 nm that is readily accessible using low-cost laser diodes, and it has cross-relaxation mechanisms between the pumped state and the ground state that produce two infrared-emitting Tm^3+^ ions for every ion activated by the pump.

**Figure 1:**
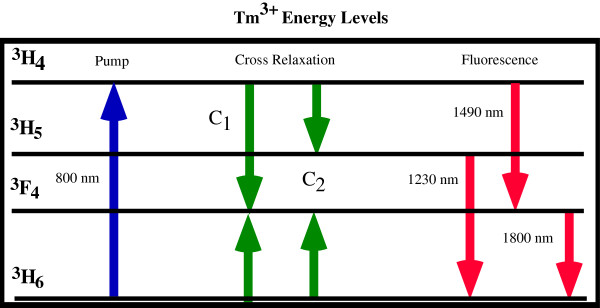
**Tm**^**3+ **^**energy levels.** Transitions for pumping, cross-relaxation, and fluorescence within the lower energy levels of Tm^3+^.

Figure 1 illustrates the lower energy levels of Tm^3+^ that become populated when the ^3^H_4_ level is pumped near 800 nm. Populations of the first three excited states can be monitored by observing infrared fluorescence near 1,400 nm from the ^3^H_4_, near 1,200 nm from the ^3^H_5_, and near 1,800 nm from the ^3^ F_4_. Two non-resonant phonon-assisted cross-relaxation mechanisms involving the pumped ^3^H_4_ state and the ground ^3^H_6_ state are also illustrated in Figure 1.

The 'two for one’ cross-relaxation process labelled C_1_ that feeds the ^3^F_4_ state is well known for Tm^3+^ and has been used in YAG [7] and YLF [8] host crystals to sensitize 2-μm sources for diode pumping. Diode-pumped lasers near 2 μm using singly doped Tm^3+^:YAG and co-doped Tm^3+^-Ho^3+^:YAG are in wide use. However, incorporating Tm^3+^ into a host crystal with reduced multi-phonon relaxation rates enables emission from the ^3^H_5_ state that is fed by the cross-relaxation process labelled C_2_. In contrast, for conventional Tm^3+^, 2-μm laser material multi-phonon quenching of the ^3^H_5_ leads to strong heat generation and distortions. Reduced multi-phonon quenching in low phonon energy materials also results in additional energy transfer processes when Tm^3+^ is co-doped with other species of rare earth ions.

This paper discusses two results that arise from Tm^3+^ cross-relaxation in low phonon energy host crystals: (1) In singly doped crystals with Tm^3+^, the C_2_ process is a phonon-assisted cross-relaxation channel that is endothermic and converts lattice phonons into infrared emission. This raises the possibility of a fundamentally new way of achieving solid-state optical cooling. (2) In crystals co-doped with Tm^3+^ and Pr^3+^, cross-relaxation results in efficient energy transfer to the lower energy levels of the Pr^3+^ ions that fluoresce in the mid-infrared. The result is an optically pumped phosphor that converts 800-nm diode light into mid-IR emission between 4 and 5.5 μm.

While these two results have different motivations, the underlying physical mechanisms are the same. Both results involve sensitized luminescence using diode-pumped Tm^3+^ ions in host crystals with reduced multi-phonon relaxation rates. The purpose of this paper is to show how crystals with low phonon energies enable these novel energy transfer processes.

## Methods

### Sensitized luminescence

In an insulator, excited-state ions can transfer energy non-radiatively to ions of the same species or of a different species through a distance-dependent electric dipole-dipole interaction [9]. The energy transfer rate between sensitizers and acceptors *W*_sa_ for two ions separated by a distance *R* can be written in the form

(1) $$W_{\mathrm{sa}}=W_{s}{\left(R_{\mathrm{cr}}/R\right)}^{6},$$

where *W*_s_ is the rate at which the sensitizer ions radiate and *R*_cr_, the critical interaction distance, is the distance between the sensitizers and acceptors for which the energy transfer rate *W*_sa_ is equal to *W*_s_. In other words, for two ions separated by the critical distance *R*_cr_, the probability of a sensitizer ion radiating is equal to the probability of its energy transfer to an acceptor ion. Therefore, crystals in which sensitizers and acceptors are on average closer than the critical radius, *W*_sa_ > *W*_s_, which results in non-radiative energy transfer being favoured over radiation.

The critical interaction distance *R*_cr_ is given by Dexter's formula [10]:

(2) $${\left(R_{\mathrm{cr}}\right)}^{6}=\left(3/4\pi \right){\left(\hbar c/\pi n\right)}^{4}Q_{a}\int \left(\frac{1}{E^{4}}\right)f_{s}^{\mathrm{ems}}\left(E\right)f_{a}^{\mathrm{abs}}\left(E\right)\mathrm{dE}.$$

In this expression, *n* is the index of refraction, *Q*_a_ is the integrated absorption cross section of the acceptor ion ∫σ(*E*)*dE*, and *f*_s_^ems^ and *f*_a_^abs^ are the normalized (∫*f*(*E*)*dE* = 1) emission and absorption spectra with *E* the photon energy equal to *ħc*/*λ*. This means that the greater the overlap between the sensitizer ion's emission spectrum and the acceptor ion's absorption spectrum, the greater the critical distance. A large critical distance allows a relatively dilute distribution of sensitizer and acceptor ions within the lattice to interact and exchange energy at rates faster than their radiative rates.

The practical consequence of Dexter’s formula is that the energy transfer is much more likely in a system in which there is significant overlap between the excited-state transitions of the sensitizing ions and the ground-state absorptions of the acceptor ions. Even in a singly doped system, in which the acceptors and sensitizers are of the same species, the pump will only interact with a small fraction of the total ions available. This means that the average distance between an excited-state ion and a ground-state ion is essentially equal to the average distance *R*_av_ between the ions in the crystal, assuming a random distribution is given by

(3) $$R_{\mathrm{av}}={\left(1/N\right)}^{1/3},$$

where *N* is the density of ions in the lattice. If *R*_av_ is less than or equal to *R*_cr_ for an interaction involving a ground-state absorption by an acceptor ion, energy transfer can occur. Interactions involving excited-state acceptor ions can usually be neglected because at pump powers of a few Watts, the average separation between these excited-state ions is usually much larger than *R*_cr_.

It is for these reasons that the cross-relaxation pathways illustrated in Figure 1 for a singly doped Tm^3+^ system are the only ones that are significant. Both C_1_ and C_2_ involve interactions between sensitizer ions excited by the pump and acceptor ions in the ground state. However, there will be no energy transfer or radiation if multi-phonon relaxation is too rapid, which is the case in many crystals that have relatively high lattice phonon energies.

### Low phonon energy crystals

Reducing the multi-phonon relaxation rates in crystalline hosts is accomplished by incorporating heavier halides, such as chlorine or bromine, which has the effect of reducing the maximum phonon energies in the crystal. As a result, electronic states with small energy gaps, corresponding to mid-IR transitions, that are normally quenched in conventional oxide and fluoride hosts will radiate in a chloride host. This effect has been used to build mid-IR rare earth-based solid-state lasers. For example, Pr^3+^:LaCl_3_ lasers have produced 5.2-μm [11] and 7.2-μm [12] emission. The LaCl_3_ host is extremely hygroscopic and offers poor mechanical stability. However, lead salts offer better mechanical stability and moisture resistance and, when created with chlorine or bromine as the halide, also have low phonon energies. For example, a room temperature 4.6-μm erbium laser using KPb_2_Cl_5_ as the crystalline host and no environmental precautions to limit exposure to moisture has been demonstrated [13]. The KPb_2_Cl_5_ host has also been used to demonstrate a Dy^3+^ 2.43-μm laser [14-16]. The success of infrared lasers using KPb_2_Cl_5_ as a host material has motivated further spectroscopic studies of Er^3+^:KPb_2_Cl_5_[17,18] in addition to other rare earth ions such as Pr^3+^[19,20] and Nd^3+^[21-24]. Activation of mid-infrared transitions of rare earth ions by reducing the phonon energies has been pushed further using KPb_2_Br_5_ as a host crystal [25,26]. This material has even lower phonon energies than KPb_2_Cl_5_ because of the substitution of Cl with the heavier Br.

### Crystal growth

Crystals with heavy halides such as chlorine have low melting points. For LaCl_3_, the melting point is 858°C; for KPb_2_Cl_5_, the melting point is 434°C; and for YCl_3_, a host crystal used in a study of cross-relaxation of singly doped thulium crystals, the melting point is 721°C. The low melting point of all these crystals allows them to be grown in fused silica ampoules in a furnace constructed of fused silica with nickel-chromium resistance wire for heating. A self-seeded vertical Bridgman can be used to grow chloride crystals from melts of anhydrous-powered starting materials under a low-pressure (approximately 100 Torr) Cl_2_ atmosphere, which is necessary to prevent the chloride compounds from disassociating. Methods for producing crystalline KPb_2_Cl_5_ and a documentation of its basic properties were reported in 1995 by Nitsch et al. [27]. Interest in incorporating rare earth ions into KPb_2_Cl_5_ has lead to further refinements of material preparation and crystal growth techniques [28-31].

Data discussed in this paper are from rare earth ions doped into two different low phonon energy crystalline hosts. YCl_3_ was chosen as a host to study Tm^3+^ cross-relaxation because TmCl_3_ and YCl_3_ share the same monoclinic crystal structure. As a result, Tm^3+^ ions incorporate at any concentration and occupy a single, highly symmetric site, which enables long excited-state lifetimes and a Stark structure that is partially resolvable even at room temperature. The KPb_2_Cl_5_ host was chosen to study singly doped crystals with Tm^3+^ or Pr^3+^ and a co-doped crystal with Tm^3+^ and Pr^3+^ because the crystal is stable under normal atmospheric conditions. In contrast, YCl_3_ crystals will dissolve in a matter of minutes when exposed to normal atmospheric humidity. For the crystal growth of KPb_2_Cl_5_, stoichiometric mixtures were prepared from anhydrous, high-purity KCl and PbCl_2_ powders mixed with small amounts of anhydrous, high-purity powders of PrCl_3_ or TmCl_3_, or both. To grow YCl_3_, anhydrous, high-purity powdered YCl_3_ and TmCl_3_ were mixed. In all cases, the powdered mixtures were melted and allowed to sit molten under approximately 100 Torr of Cl_2_ for several hours to reduce oxide impurities. The melt, contained in a 10-mm inner diameter fused silica ampoule with a tapered tip, was cooled over a period of 5 days while remaining under the Cl_2_ atmosphere. The finished samples were polycrystalline with large grains and were un-oriented.

### Spectroscopy

Unpolarized fluorescence spectra between 1,600 and 5,500 nm were collected with a 0.20-m monochrometer. Fluorescence was induced with laser diodes gated to produce 50-ms pulses. The diode pump powers were between 0.25 and 2.0 W. A pulse repetition rate of 10 Hz was used to synchronize a lock-in amplifier that received its input from a photo-detector mounted at the exit slits of the monochrometer. Spectra were collected using three passes - one for the 1,100- to 1,700-nm range, one for the 1,550- to 3,000-nm range, and one for the 3,000- to 5,500-nm range. An InGaAs photo-detector was used for the 1,100- to 1,700-nm range. For the other two spectral ranges that covered 1,550 to 5,500 nm, a liquid nitrogen-cooled InSb was used for photo-detection. For the 3,000- to 5,500-nm range, a long pass filter that blocked wavelengths less than 2,500 nm was in place to eliminate the short wavelength features from appearing in higher order. Also, for spectral acquisition at wavelengths greater than 2,500 nm, the monochrometer was purged with dry nitrogen gas in order to reduce a strong absorption feature at 4,300 nm resulting from atmospheric CO_2_. Emission was measured with the Tm^3+^:YCl_3_ remaining sealed in the fused silica ampoules to prevent degradation from exposure to atmospheric moisture. Fused silica is transparent for the range of emission wavelengths studied. For Tm^3+^:KPb_2_Cl_5_, no environmental precautions were used. In each case, the wavelength dependence of the complete light collection and detection system was calibrated using a blackbody source. Spectra were corrected using the system response function obtained from the blackbody calibration. To observe fluorescent decays, the laser diodes were operated in pulsed mode to pump the ^3^H_4_ level of Tm^3+^, and a digitizing oscilloscope recorded the transient response from the photo-detectors. During fluorescent decay measurements, the monochrometer acted as a filter to isolate emission at wavelengths associated with specific energy levels.

## Results and discussion

### Spectroscopy of singly doped Tm^3+^ crystals

**Figure 2:**
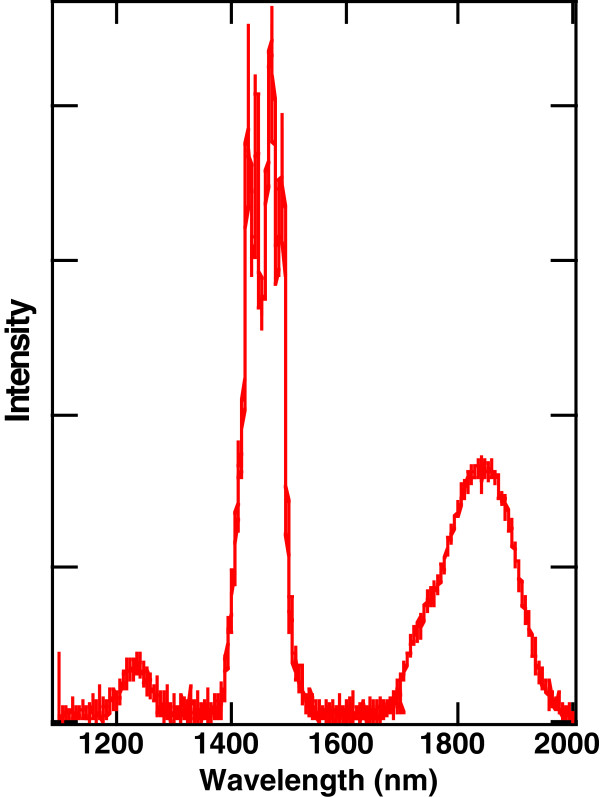
**Fluorescence from Tm**^**3+**^**:KPb**_**2**_**Cl**_**5**_**.** Fluorescence spectrum at 300 K between 1,100 and 2,000 nm of Tm^3+^:KPb_2_Cl_5_ that results from pumping with an 805-nm laser diode.

Figure 2 shows a fluorescence spectrum at 300 K between 1,100 and 2,000 nm of Tm^3+^:KPb_2_Cl_5_ that results from pumping with a 1.5-W, 805-nm laser diode [32]. The spectrum has three features that are typical of Tm^3+^ spectra in low phonon energy hosts. The emissions originate from the first three excited states and arise from the following transitions: ^3^H_4_ → ^3^ F_4_ (centred at 1,450 nm), ^3^H_5_ → ^3^H_6_, (centred at 1,250 nm), and ^3^ F_4_ → ^3^H_6_ (centred at 1,850 nm). The emissions at 1,450 and 1,250 nm are a characteristic of Tm^3+^ in a low phonon energy host crystal. For Tm^3+^ in YAG or YLF, these emissions are quenched by multi-phonon relaxation, and the only IR emission observed is the broadband centred at 1,850 nm arising from the ^3^F_4_ level.

**Figure 3:**
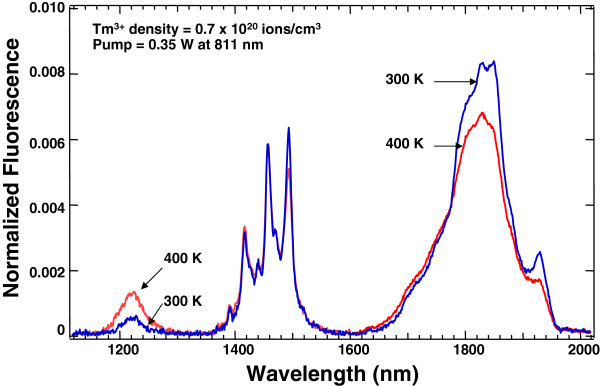
**Fluorescence from Tm**^**3+**^**:YCl**_**3**_**.** Comparison of fluorescence spectrum at 300 and 400 K between 1,100 and 2,000 nm of Tm^3+^:YCl_3_ that results from pumping with an 811-nm laser diode.

**Figure 4:**
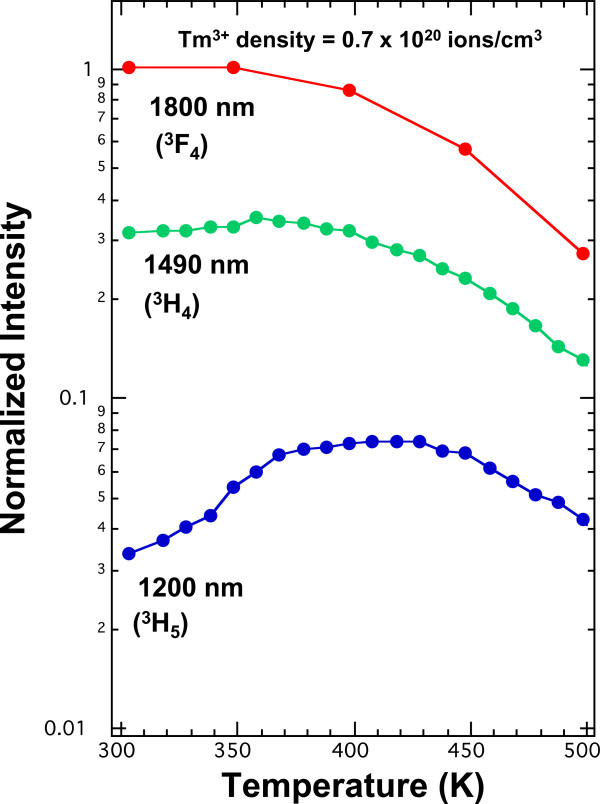
**Temperature dependence of infrared fluorescence from Tm**^**3+**^**:YCl**_**3**_**.** Normalized fluorescence intensity versus temperature between 300 and 500 K for Tm^3+^:YCl_3_. The fluorescence intensity of the ^3^ F_4_ level at 300 K is normalized to 1. The sample has a Tm^3+^ concentration of 0.7 × 10^20^ ions/cm^3^.

For the same 1,100- to 2,000-nm spectral range, a fluorescence spectrum from Tm^3+^:YCl_3_ at 300 K arising from pumping with a 0.35-W, 811-nm laser diode is shown in Figure 3[33]. Because YCl_3_ is also a low phonon energy host, the same three spectral features appear. In YCl_3_, the Tm^3+^ ions are at sites with higher symmetry than in KPb_2_Cl_5_. As a result, more of the Stark multiplet structure is resolvable in the emission lines in Figure 3 than in Figure 2. Also shown in Figure 3 is an overlap of a fluorescence spectrum at 400 K from the same crystal under the same pump conditions. As the temperature is increased, there is a small reduction in emission at 1,850 nm from the ^3^F_4_ level, but a doubling in 1,250-nm emission from the ^3^H_5_ level. The increase in emission from the ^3^H_5_ as the temperature is raised is an interesting and counterintuitive result. The effect of a temperature increase on this emission is illustrated more graphically in Figure 4[33]. It shows the normalized fluorescence intensity at three specific wavelengths as a function of temperature between 300 and 500 K. The wavelengths chosen reflect the populations of the first three excited states for Tm^3+^. The data show that the population of ^3^H_5_ state increases relative to the other states as the temperature rises.

### Cross-relaxation in singly doped Tm^3+^ crystals

The anomalous behaviour of the 1,200-nm fluorescence from the ^3^H_5_ state can be explained as arising from phonon-assisted cross-relaxation [34]. The processes illustrated in Figure 1 labelled C_1_ and C_2_ are both non-resonant and require phonon assistance to complete. C_1_ is the process already known in Tm^3+^-doped YAG and YLF that involves an interaction between a ^3^H_4_ ion activated by the pump and a ^3^H_6_ ion in the ground state to produce two ^3^F_4_ ions. The C_1_ process results in an excess of energy that must be converted to phonons. The energy gap for this cross-relaxation is 698 cm^-1^, which corresponds to the creation of about three phonons in the YCl_3_ lattice that has a maximum phonon energy of 260 cm^-1^.

For the process C_2_ that feeds both the ^3^F_4_ and ^3^H_5_ levels, the energy gap is a deficit of -641 cm^-1^. This process must absorb three phonons from the lattice to complete. However, phonon absorption processes have much stronger temperature dependence than phonon-emitting processes. At low temperatures, any relaxation process that emits phonons, such as cross-relaxation or multi-phonon relaxation, can proceed through spontaneous emission. At high temperatures, stimulated emission will occur as phonon occupation increases, which increases the relaxation rate. Therefore, the temperature dependence of the rate for a phonon emission process *W*_e_ is given by

(4) $$W_{e}\left(T\right)=W_{e}\left(T=0\right){\left(1+n\right)}^{N_{e}},$$

where *N*_e_ is the number of phonons (Δ*E*/*ħω*) emitted to fill the energy gap Δ*E* that have energy *ħω* and *n* is the phonon occupation number [35].

However, phonon absorption processes must have occupied phonon states in order to proceed. The temperature dependence of the rate *W*_a_ for a phonon absorption process is given by

(5) $$W_{a}\left(T\right)=W_{a}\left(T=0\right){\left(n\right)}^{N_{a}},$$

where *N*_a_ is the number of phonons absorbed. The temperature dependencies of Equations 4 and 5 arise because the phonon occupation number *n* follows a Bose-Einstein distribution given by

(6) $$n={\left(\exp\left[\hbar \omega /\mathrm{kT}\right]-1\right)}^{-1},$$

where *ħω* is the maximum phonon energy (260 cm^-1^ for YCl_3_) [36]. Therefore, the maximum phonon energy is the most important parameter in controlling the temperature and energy gap dependence of all phonon-assisted relaxation processes, including cross-relaxation and multi-phonon relaxation.

Excited state populations and lifetimes for Tm^3+^, which ensue after pumping the ^3^H_4_ state at 800 nm, depend on the competition between spontaneous emissions of radiation, cross-relaxation, multi-phonon relaxation, and up-conversion. At temperatures greater than 500 K, multi-phonon relaxation is the dominant process, which results in quenching of the fluorescence from all levels. At room temperature, near 300 K, multi-phonon relaxation is reduced and cross-relaxation can proceed. However, at 300 K, the occupation of phonon states is still substantial, which allows the endothermic process C_2_ to compete with the exothermic process C_1_.

A macroscopic model of the populations of the four lowest levels of Tm^3+^ was constructed using coupled time-dependent rate equations [33]. Rate constants for spontaneous emission, cross-relaxation, and up-conversion were determined by fitting the model to fluorescence lifetime data at 300 K, a temperature at which multi-phonon relaxation can be neglected. Rate constants for multi-phonon relaxation were determined by fitting the model to lifetime data above 400 K, temperatures at which multi-phonon relaxation is significant [33].

**Figure 5:**
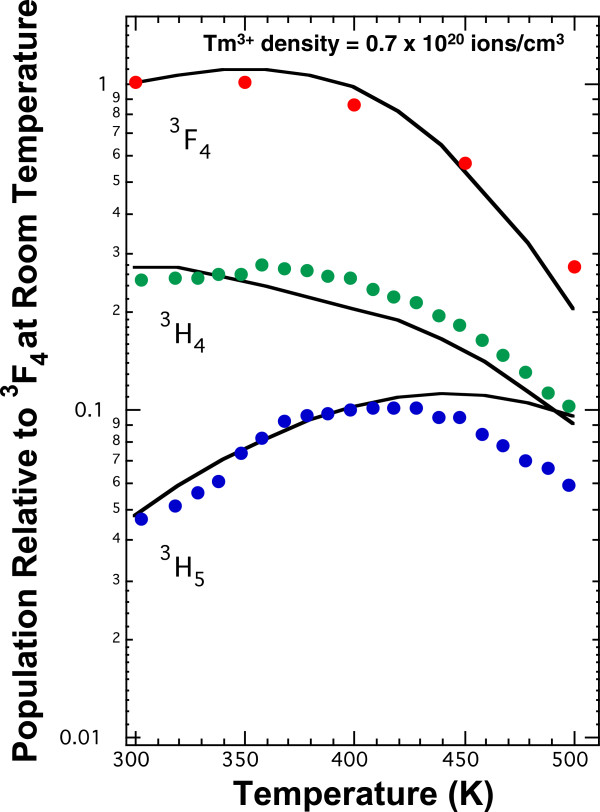
**Temperature dependence of infrared fluorescence from Tm**^**3+**^**:YCl**_**3**_**.** Overlay of temperature-dependent rate model for the relative population of the three lower levels for Tm^3+^:YCl_3_ with the temperature-dependent intensity data from Figure 4. The solid lines are the model, and the markers are the data. The population of the ^3^F_4_ level at 300 K is normalized to 1. The sample has a Tm^3+^ concentration of 0.7 × 10^20^ ions/cm^3^.

After the determination of the rate constants, the relative populations of the three lower levels of Tm^3+^ as a function of temperature were determined using the rate model and the temperature dependencies of Equations 4 and 5 for cross-relaxation processes C_1_ and C_2_, respectively. Figure 5 shows an overlay of the temperature-dependent rate modelling with the temperature-dependent intensity data from Figure 4[33]. The model predicts the observed increase in emission from the ^3^H_5_ level as the temperature is raised. The model shows that the branching ratio for the ^3^H_4_ to ^3^H_5_ transition is less than 1%, and as a result, the population of the ^3^H_5_ arises almost entirely from the C_2_ cross-relaxation process [33]. Between 300 and 400 K the model also predicts the observation that the emission from the ^3^F_4_ and ^3^H_4_ levels is unchanged as the temperature rises because multi-phonon relaxation has not increased to a level that it competes with radiation and cross-relaxation.

This result is significant because it implies that the process C_2_ converts lattice phonons into 1,200-nm radiation, which is a cooling effect. In contrast to previous demonstrations of solid-state optical cooling from anti-Stokes emission [37-43], cooling from cross-relaxation will not lose efficiency at low temperatures because the -641 cm^-1^ energy gap for the process is temperature independent. At low-temperatures, cooling from anti-Stokes emission loses efficiency because of thermal depopulation of the upper Stark levels.

Also of interest for Tm^3+^:YCl_3_ is that additional study of the concentration dependence of the cross-relaxation rates determined that the critical radius *R*_cr_ at room temperature for the energy transfer is about 15 Å. That distance is comparable to *R*_cr_ for Tm^3+^ cross-relaxation in conventional oxide and fluoride hosts [7,8]. This implies that the endothermic cross-relaxation process C_2_ is enabled by the reduction in multi-phonon quenching and not because interaction rates between neighbouring Tm^3+^ ions are changed significantly by a chloride host. These spectroscopic results suggest that a heat generation study should be conducted for the near-IR-pumped Tm^3+^ in a low phonon energy host.

### Energy transfer in Tm^3+^-Pr^3+^ co-doped crystals

In addition to its own IR-emitting properties, the Tm^3+^ ion has been used to sensitize other rare earth ions for diode pumping. Most notable is the Ho^3+^ ion, which has a useful IR laser transition at 2.1 μm from its first excited state to its ground state but lacks a level that absorbs at 800 nm. Energy transfer from Tm^3+^ to Ho^3+^ has been used to create diode-pumped 2.1-μm lasers using YLF [7] and YAG [8] host crystals. Tm^3+^ sensitization has also been used in low phonon energy crystals. For example, Nd^3+^ has absorption near 800 nm. However, for co-doped Tm^3+^-Nd^3+^:KPb_2_Cl_5_, the presence of the Tm^3+^ is known to increase the absorption of the pump and enhance the IR emission from the Nd^3+^ ions [44]. An additional example is co-doped Tm^3+^-Pr^3+^:CsCdBr_3_, in which pumping the ^3^H_4_ level of Tm^3+^ results in energy transfer and up-conversion to emitting states in the visible [45].

**Figure 6:**
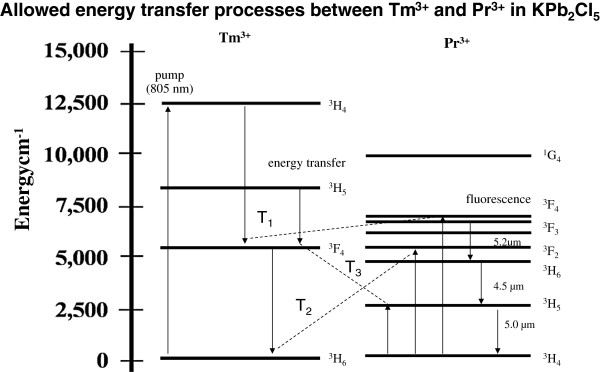
**Energy transfer processes for co-doped Tm**^**3+**^**-Pr**^**3+**^**:KPb**_**2**_**Cl**_**5**_**.** The first three excited states of Tm^3+^-sensitizing ions are all resonant with ground-state transitions of Pr^3+^ acceptor ions.

Energy transfer from the ^3^H_4_ state of Tm^3+^ to the IR-emitting states of Pr^3+^ in a low phonon energy host crystal is also an interesting phenomenon. Like Ho^3+^, the Pr^3+^ ion also lacks absorption at 800 nm. However, transitions out of the first three excited states of Tm^3+^ that populate through cross-relaxation are resonant with absorption transitions out of the Pr^3+^ ground state to excited states of Pr^3+^ that radiate in the mid-IR. Figure 6 compares the lower energy levels of Tm^3+^ to the lower levels of Pr^3+^ and illustrates three possible pathways for resonant energy transfer that involve excited-state Tm^3+^-sensitizing ions interacting with ground-state Pr^3+^ acceptor ions.

**Figure 7:**
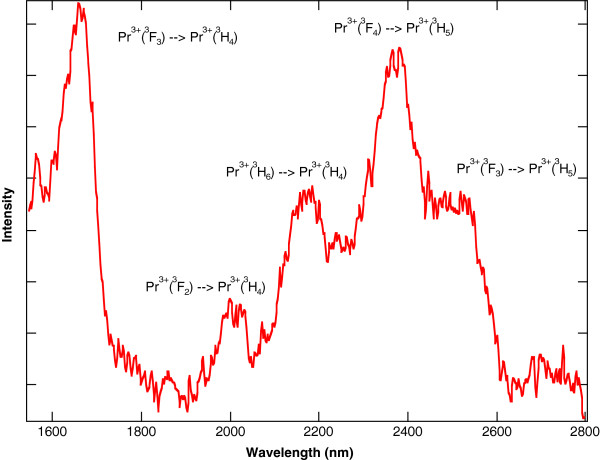
**Fluorescence from 1,600 to 2,800 nm resulting from 1,483-nm pumping of Pr**^**3+**^**:KPb**_**2**_**Cl**_**5**_**.** The sample has a Pr^3+^ concentration of 1.5 × 10^20^ ions/cm^3^.

**Figure 8:**
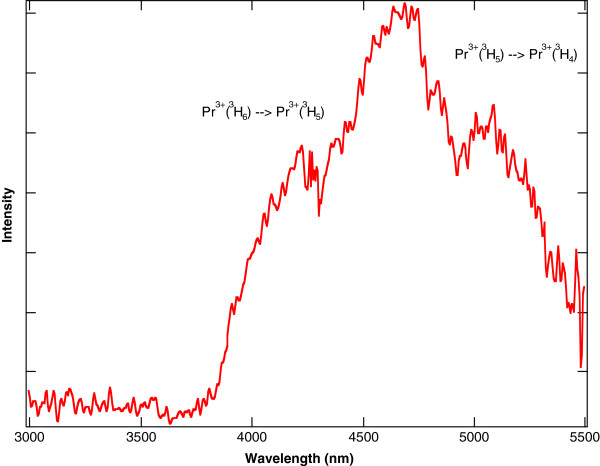
**Fluorescence from 3,000 to 5,500 nm resulting from 1,483-nm pumping of Pr**^**3+**^**:KPb**_**2**_**Cl**_**5**_**.** The sample has a Pr^3+^ concentration of 1.5 × 10^20^ ions/cm^3^.

In contrast to Pr^3+^:YAG or Pr^3+^:YLF, Pr^3+^ ions in a chloride host crystal will radiate at mid-IR wavelengths because the lower energy levels are no longer quenched by multi-phonon relaxation. This effect was exploited to make 5.2- and 7.2-μm lasers using Pr^3+^:LCl_3_[11,12]. For Pr^3+^ doped into KPb_2_Cl_5_, the lower energy levels will also radiate in the mid-IR. The mid-IR fluorescence can be observed in singly doped Pr^3+^:KPb_2_Cl_5_ when the ^3^F_4_ level is pumped directly with a 1.5-W, 1,483-nm laser diode. For Pr^3+^:KPb_2_Cl_5_ under this pump, the room temperature fluorescence that results from 1,600 to 2,800 nm is shown in Figure 7 and from 3,000 to 5,500 nm is shown in Figure 8[32]. Each feature in the spectra is labelled with the associated Pr^3+^ energy level transition.

**Figure 9:**
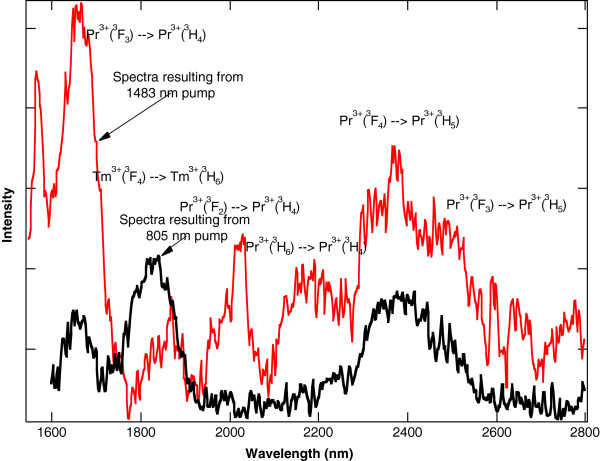
**Fluorescence from 1,600 to 2,800 nm from Tm**^**3+**^**-Pr**^**3+**^**:KPb**_**2**_**Cl**_**5**_**.** Fluorescence resulting from 1,483-nm pumping of Tm^3+^-Pr^3+^:KPb_2_Cl_5_ compared to fluorescence resulting from 805-nm pumping. The sample has a Pr^3+^ concentration of 1.5 × 10^20^ ions/cm^3^ and a Tm^3+^ concentration of 3.0 × 10^20^ ions/cm^3^.

**Figure 10:**
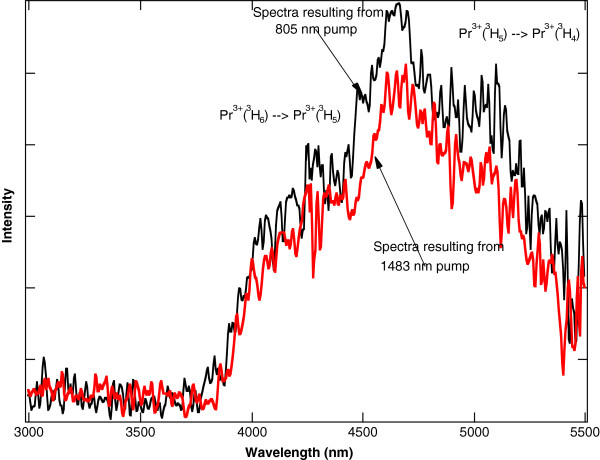
**Fluorescence from 3,000 to 5,500 nm from Tm**^**3+**^**-Pr**^**3+**^**:KPb**_**2**_**Cl**_**5**_**.** Fluorescence resulting from 1,483-nm pumping of Tm^3+^-Pr^3+^:KPb_2_Cl_5_ compared to fluorescence resulting from 805-nm pumping. The sample has a Pr^3+^ concentration of 1.5 × 10^20^ ions/cm^3^ and a Tm^3+^ concentration of 3.0 × 10^20^ ions/cm^3^.

The Tm^3+^ sensitization of Pr^3+^:KPb_2_Cl_5_ allows for more convenient 800-nm diode pumping. For a co-doped Tm^3+^-Pr^3+^:KPb_2_Cl_5_ crystal using a 1.5-W, 805-nm laser diode as a pump source, the same broadband mid-IR emission between 4,000 and 5,500 nm from the Pr^3+^ ions is observed. The room temperature fluorescence that results from 805-nm pumping of the co-doped crystal overlapped with the fluorescence that results from the 1,483-nm pumping of the same co-doped crystal from 1,600 to 2,800 nm is shown in Figure 9 and from 3,000 to 5,500 nm is shown in Figure 10[32]. The overlap of the two spectra in Figure 10 show that the broadband emission between 4,000 and 5,500 nm from the Pr^3+^ ions is essentially the same for the two crystals, even though the pump sources are at different wavelengths. As expected, Figure 9 shows less emission at 2,400 nm out of the ^3^F_4_ state of Pr^3+^ in the co-doped crystal compared to 1,483-nm pumping of the singly doped crystal, because the ^3^F_4_ state of Pr^3+^ is no longer being pumped directly.

**Figure 11:**
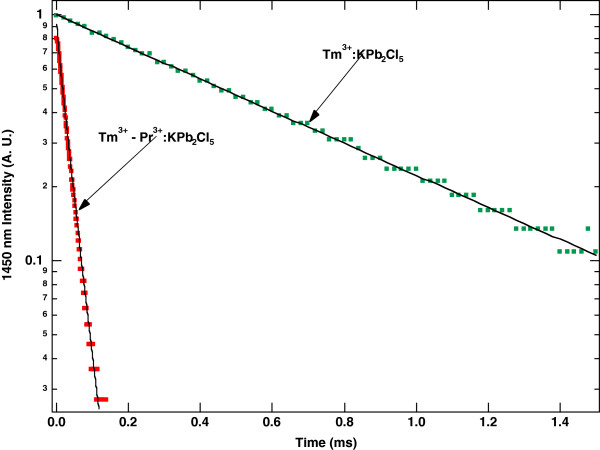
**Transient decays from the **^**3**^**H**_**4 **_**level of Tm**^**3+**^**.** Room temperature normalized fluorescence decays from the ^3^H_4_ level of Tm^3+^ arising from 805-nm diode pumping. Comparison is made of 1,450-nm emission from Tm^3+^:KPb_2_Cl_5_ to 1,450-nm emission from Tm^3+^-Pr^3+^:KPb_2_Cl_5_.

Figure 11 shows lifetime data for the 1,450-nm emission from the ^3^H_4_ level of Tm^3+^ resulting from 805-nm pumping for the singly doped and co-doped samples [32]. Comparison of the 1,450-nm emission from Tm^3+^:KPb_2_Cl_5_ to 1,450-nm emission from Tm^3+^-Pr^3+^:KPb_2_Cl_5_ shows the rapid quenching of emission from the Tm^3+^ because of energy transfer to the Pr^3+^. Analyses of the Tm^3+^ lifetime data for the co-doped crystal show that the energy transfer processes from the Tm^3+^ sensitizers to the Pr^3+^ acceptors have high quantum efficiencies. For the energy transfer process labelled T_1_ in Figure 6, the quantum efficiency *η*_1_ is estimated at 94%, and for the process labelled T_2_ in Figure 6, the estimated quantum efficiency *η*_2_ is 83% [32]. The process labelled T_3_ can be neglected because the ^3^H_5_ level of Tm^3+^ never obtains significant population. Further analysis of the decay transients provides estimates of 11 and 12 Å, respectively, for the critical radii of energy transfer from the ^3^H_4_ and ^3^F_4_ states of Tm^3+^. The critical radii for this co-doped system are comparable to the critical radii of electric dipole-dipole interactions involving rare earth ions in other host crystals, such as the cross relaxation of Tm^3+^ in YCl_3_ discussed in the earlier part of this paper.

The usefulness of this system is that it functions as an optically pumped mid-IR phosphor that converts light from 805-nm diodes to broadband mid-IR from 4 to 5.5 μm. The 805-nm diode sources are low in cost compared to 1.5- or 2-μm sources that would activate the Pr^3+^ mid-IR emission directly. This material could be used as a low-cost method for detecting gases with absorptions in the 4- to 5.5-μm range. For example, the small dip at 4,300 nm in the fluorescence spectra shown in Figure 10 is an artefact of a less than perfect purge of the optical path with dry nitrogen. Without the purge, the 4,300-nm fluorescence emitted by the diode-pumped crystal is completely absorbed by atmospheric CO_2_. In effect, the experimental setup functioned as a very sensitive atmospheric CO_2_ detector.

## Conclusions

This paper discussed two applications of Tm^3+^ sensitization of rare earth-doped low phonon energy host crystals, in which the resulting reduction in multi-phonon relaxation rates enables useful energy transfer processes to occur that are quenched in conventional oxide and fluoride crystals. One application is the enabling of an endothermic cross-relaxation process for Tm^3+^ that converts lattice phonons to infrared emission near 1,200 nm. The existence of this process suggests that endothermic phonon-assisted energy transfer could be a fundamentally new way of achieving optical cooling in a solid. The other application is a novel optically pumped mid-IR phosphor that converts 805-nm light from readily available low-cost diodes into broadband emission from 4 to 5.5 μm. The phosphor is efficient, low-cost, and scalable.

Application of theories for electric dipole-dipole sensitizer-acceptor interactions shows that the critical radii for energy transfer processes between rare earth ions do not change significantly between various host crystals. The novel energy transfer processes observed in low phonon energy host crystals occur because the multi-phonon relaxation rates for the levels involved are reduced and no longer compete with the radiative and non-radiative energy transfer rates. In imagining new kinds of applications for low phonon energy crystals, circumstances in which the multi-phonon relaxation rates can be reduced to much less than the known rates for electric dipole interactions should be investigated.

## Competing interests

The authors declare that they have no competing interests.

## Authors’ contributions

JG drafted the manuscript, prepared the samples, and participated in acquiring, analyzing and interpreting the data and in conceiving and designing these experiments. SRB participated in acquiring, analyzing, and interpreting the data, in conceiving and designing these experiments, and in revising the manuscript. Both authors read and approved the final manuscript.
